# Integrated Avoid Collision Control of Autonomous Vehicle Based on Trajectory Re-Planning and V2V Information Interaction

**DOI:** 10.3390/s20041079

**Published:** 2020-02-17

**Authors:** Fen Lin, Kaizheng Wang, Youqun Zhao, Shaobo Wang

**Affiliations:** College of Energy and Power Engineering, Nanjing University of Aeronautics and Astronautics, Nanjing 210016, China; kzwang.nuaa@gmail.com (K.W.); yqzhao@nuaa.edu.cn (Y.Z.); shaobo@nuaa.edu.cn (S.W.)

**Keywords:** autonomous vehicle, avoidance collision control, trajectory re-planning, obstacle trajectory prediction, V2V

## Abstract

An integrated longitudinal-lateral control method is proposed for autonomous vehicle trajectory tracking and dynamic collision avoidance. A method of obstacle trajectory prediction is proposed, in which the trajectory of the obstacle is predicted and the dynamic solution of the reference trajectory is realized. Aiming at the lane changing scene of autonomous vehicles driving in the same direction and adjacent lanes, a trajectory re-planning motion controller with the penalty function is designed. The reference trajectory parameterized output of local reprogramming is realized by using the method of curve fitting. In the framework of integrated control, Fuzzy adaptive (proportional-integral) PI controller is proposed for longitudinal velocity tracking. The selection and control of controller and velocity are realized by logical threshold method; A model predictive control (MPC) with vehicle-to-vehicle (V2V) information interaction modular and the driver characteristics is proposed for direction control. According to the control target, the objective function and constraints of the controller are designed. The proposed method’s performance in different scenarios is verified by simulation. The results show that the autonomous vehicles can avoid collision and have good stability.

## 1. Introduction

The autonomous vehicle is an emerging technology that provides a safe and efficient transportation experience. As part of the intelligent transportation system, it has a very wide application prospect [1,2]. The autonomous vehicle refers to a class of vehicles that can carry out sensing and decision-making, track planning, and tracking. Trajectory tracking control is the basic problem and necessary condition of autonomous vehicle research [3]. Due to the application of advanced sensing technology and the development of vehicle state estimation algorithm, the state information of the vehicle becomes more observable and more accurate, such as tire force conditions, sideslip angle, yaw rate, which are closely related to vehicle handling stability [4,5,6], even the coefficient of friction of tires [7]. 

There has been a great deal of research on path planning and tracking control algorithms, such as inverse kinetic compensation feedback control [8], the optimization algorithm based on the sampling fusion and quadratic programming model [9], the linear combination method using weighted cost function [10], integrated local path planning and tracking control [11], using non-linear programming model [12] and so on. As the vehicle has its own mechanical structure, stability, driver handling capacity and other restrictions, it is not easy to apply these methods directly to the collision scene of the vehicle. Therefore, for the path planning problems, the movement states of other vehicles need to be considered to solve the collision problem on the road. In order to improve the active safety performance of the vehicle and reduce the occurrence rate of rear-end collision accident, research and development of high-performance vehicle collision avoidance system has become an urgent need. Auto active collision avoidance system which using modern information and sensing technology to obtain outside information takes a various information of vehicle and traffic into account to identify the collision risk. The path planning of the vehicle obstacle avoidance system is to establish a collision-free trajectory. The geometric characteristics of the obstacle and the movement constraints of the autonomous vehicle are considered [13]. Literature [14] proposed a comprehensive autonomous vehicle collision avoidance system framework, using an improved harmonic velocity potential method for path planning, and using fuzzy adaptive module to maintain a safe distance. The simulation and verification of the method are carried out in the curve scene with low traffic flow. In literature [15], a framework is proposed to keep the collision-free path planning and tracking. The trigonometric function of the road and the exponential function of the obstacle are constructed by the three-dimensional virtual dangerous potential field, and the path is tracked by the multi-constrained model predictive control (MMPC). In the literature [16], an alternative control framework for integrated local path planning and path tracking using Model Predictive Control (MPC) is proposed. The two packages are proposed for vehicle stability and obstacle avoidance functions, respectively, and have been successfully tested on the test vehicle. In the literature [17], a rolling time domain control method for autonomous vehicle obstacle avoidance motion planning in uncertain dynamic environment is proposed. This method deals with perturbation effects and input and state constraints based on the structure of set theory. The goal of any collision avoidance system is to design a control algorithm to avoid an imminent accident. Longitudinal control (emergency braking) and lateral control (active steering) are possible ways to avoid collisions. The longitudinal control method is limited by the longitudinal distance between the vehicles, in which case the active lateral movement is more appropriate to avoid obstacles.

In order to achieve the obstacle avoidance function, the autonomous vehicle needs to obtain the obstacle information through detectors such as radar detectors. Before lane change, it is necessary to expand the distance from the obstacle to avoid the collision. In the implementation of obstacle avoidance function, there are several ways to control the front wheel angle and the velocity of the vehicle to choose. In this paper, the driving characteristics of the driver are added to the controller n-order to meet the manipulation of human drivers and habits. A trajectory re-planning controller is constructed by using the 5-order polynomial fitting algorithm.

With the development of vehicle-to-vehicle (V2V) communication technology, drivers, vehicles and road information can be shared between vehicles [18]. The intention of the driver, the state information of the vehicle such as sideslip angle, intersection lights, and road condition information are available. Therefore, more information is used to ensure the safety of the vehicle. In addition to the state of the vehicle itself, other vehicle states and road information are required to achieve safe driving [19,20]. In recent years, obstacle avoidance algorithms based on V2V communication have been widely studied. In literature [21], the vehicle information network is established to obtain the vehicle states during the lane change. Using the information to establish the vehicle dynamics model, calculate the expected acceleration value to control the vehicle throttle and brake pedal, in order to achieve the road on the road when the vehicle collision avoidance. In literature [22], from the point of view of driving comfort, through the vehicle communication system, the front vehicle information is obtained to calculate the ideal vehicle spacing, and complete the cooperative initiative collision avoidance with the method of allocating acceleration to the front and rear vehicles. In literature [23], a cooperative collision avoidance system (CCA) based on V2V communication system is proposed, which can effectively avoid the collision and ensure the driver’s comfort. In literature [24], a decentralized autonomous vehicle collaborative transformation lane decision framework is proposed. The influence of decision frame on traffic stability, efficiency, uniformity, and safety was studied. In summary, with the continuous development of Cooperative Vehicle Infrastructure System (CVIS), information of driver, vehicle and road can be shared between vehicles. Based on the information interaction between vehicles, it is a trend to realize vehicle trajectory tracking and active collision avoidance by optimal processing of intelligent algorithm. However, in most articles, only the information of vehicle obtained from V2V communication at the moment k is used as obstacle avoidance information, ignoring the collision risk that the vehicle still has at the moment k+1. In this paper, the vehicle information interactive model is presented, so that more interactive information is used to optimize trajectory planning. With the information interaction model presented, the vehicle real-time position information and its predicted trajectory can be used as reference obstacle information to realize dynamic obstacle avoidance.

Nonlinearity, time variability is a typical feature of autonomous vehicles [25]. MPC has low accuracy requirements for the model and it is very robust to the time-varying characteristics of the system model [26]. At the next moment, if the longitudinal position and lateral position of the vehicle can be predicted, the risk of collision can be effectively reduced. Predicting the location of a vehicle requires a large amount of information to be processed at the same time, including driver’s intent, vehicle states, environmental states, various restrictions, and path planning. MPC can effectively solve the multi-objective optimization problem while effectively reducing the burden of calculation [27,28,29]. In this paper, The MPC controller is proposed to process the vehicle interactive information. That is, the state information of the two vehicles is processed by the MPC controller at the same time to realize the path tracking and active collision avoidance of the autonomous vehicle.

The contributions of this paper mainly include (1) an integrated avoid collision control framework is proposed for autonomous vehicle to solve the safety problem of adjacent lane exchanging vehicles. The interactive information is obtained by the vehicle as the state variables of the controller. (2) A moving obstacle trajectory prediction algorithm is presented. The obstacle avoidance function is realized by the trajectory re-planning controller embedded in driver’s characteristics. (3) An MPC is proposed as a lateral controller, and the fuzzy adaptive PI control algorithm is proposed for longitudinal control of the vehicle to achieve multi-objective problem optimization and obstacle avoidance function.

The rests of this paper are organized as follows. In Section 1, the vehicle model, driver model to form the system dynamic model are described. Obstacle trajectory prediction model is also presented. In Section 2, the main contributions of this paper, including the trajectory re-planning control framework for lane exchanging of two vehicles, the fuzzy adaptive PI longitudinal controller design and analysis to enable the accurate velocity tracking, the MPC with V2V information interaction modular and the driver characteristics is proposed for direction control. In Section 3, the proposed method’s performance in different scenarios are verified according simulation. These scenarios include different initial vehicle distances, vehicle constant velocity, and variable velocity. The conclusions are presented in Section 4.

## 2. System Modeling

In this section, the vehicle model, the driver model, and the moving obstacle trajectory prediction model are described. The vehicle model and the driver model are integrated to establish a nonlinear dynamic model considering the drivers’ handling characteristics. In the following model, the superscript i=p,q is used to represent the two vehicles with different states, respectively. The multi-vehicle interactive state information is processed by the vehicle p, and the interactive state information is supplied by the vehicle q.

### 2.1. Vehicle Model

**Figure 1:**
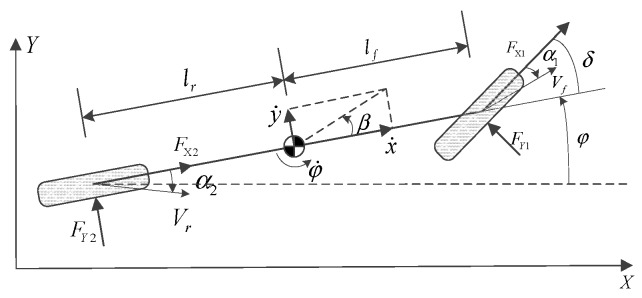
Vehicle model.

It is of great significance to establish a suitable vehicle dynamics model for the MPC controller [30]. Simplifying the vehicle dynamics model not only reflects the basic dynamic characteristics of the vehicle, but also ensures the real-time performance of the control algorithm. A bicycle model, as shown in Figure 1, is used to describe the dynamics of the two vehicles, including longitudinal, yaw, and lateral motions [31].

Assuming that the front tire steering angle is small and the dynamics and parameters of the two vehicles are the same. In the global coordinates oxy, $F_{xf}$ and $F_{xr}$ are the longitudinal forces acting on the front and rear tires, respectively. ${\dot{x}}^{i}$ and ${\dot{y}}^{i}$ are the longitudinal and lateral velocity of the vehicle, respectively. $l_{f}$ and $l_{r}$ are the distance from the center of gravity of the vehicle to the front and rear axles, respectively. ${\dot{\varphi }}^{i}$ is the yaw rate of the vehicle. m is the vehicle mass. $I_{Z}$ is the yaw moment of inertia of the vehicle. 

From Figure 1, dynamics of the vehicle can be described as follows:

(1) $$m{\ddot{x}}^{i}=m{\dot{y}}^{i}{\dot{\varphi }}^{i}+F_{x1}^{i}+F_{x2}^{i}$$

(2) $$m{\ddot{y}}^{i}=-m{\dot{x}}^{i}{\dot{\varphi }}^{i}+F_{y1}^{i}+F_{y2}^{i}$$

(3) $$I_{z}{\ddot{\varphi }}^{i}=l_{f}F_{y1}^{i}-l_{r}F_{y2}^{i}$$

In the process of vehicle lane change, the direction angle of the vehicle is very small, that is to meet the following approximate conditions, $\cos({\dot{\varphi }}^{i})\approx 1$, $\sin({\dot{\varphi }}^{i})\approx {\dot{\varphi }}^{i}$, convert as follows:

(4) $${\dot{X}}^{i}={\dot{x}}^{i}-{\dot{y}}^{i}{\dot{\varphi }}^{i}$$

(5) $${\dot{Y}}^{i}={\dot{x}}^{i}{\dot{\varphi }}^{i}+{\dot{y}}^{i}$$

where ${\dot{X}}^{i}$ and ${\dot{Y}}^{i}$ are longitudinal and lateral positions of the vehicle along the global coordinates, X and Y, respectively.

The front and rear lateral tire forces can be written as the functions of the tire slip angles described as follows:

(6) $$F_{y1}^{i}=C_{cf}\alpha_{1}^{i}\;\;\;\;\;F_{y2}^{i}=C_{cr}\alpha_{2}^{i}$$

where $C_{cf}$ and $C_{cr}$ are the front and rear tire cornering stiffness and $\alpha_{1}$ are $\alpha_{2}$ the front and rear tire slip angle.

(7) $$\alpha_{1}^{i}=\delta_{f}^{i}-\frac{l_{f}{\dot{\varphi }}^{i}}{{\dot{x}}^{i}}-\beta^{i}\;\;\;\;\;\alpha_{2}^{i}=\frac{l_{r}{\dot{\varphi }}^{i}}{{\dot{x}}^{i}}-\beta^{i}$$

where $\delta_{f}^{i}$ is the front wheel steering angle and the vehicle side slip angle, $\beta^{i}={\dot{y}}^{i}/{\dot{x}}^{i}$.

The front and rear longitudinal tire forces can be written as the functions of the tire slip rate described as follows:

(8) $$F_{x1}^{i}=C_{lf}s_{1}^{i}\;\;\;\;\;F_{x2}^{i}=C_{lr}s_{2}^{i}$$

where $C_{lf}$ and $C_{lr}$ are the front and rear tire longitudinal stiffness and $s_{1}^{i}$ are $s_{2}^{i}$ the front and rear tire slip rate.

### 2.2. Human Driver Model

The driver model is essentially a physical equation that simulates the driver’s behavior. In the study of the driver-vehicle system closed-loop system, the driver plays a “controller” role and the driver adjusts the direction according the characteristics. In this paper, the driver model and the vehicle model are integrated to establish the driver-vehicle closed-loop system. The young and aged driver’s handling characteristics, including advance time, delay time, and steering wheel angle ratio, are evaluated, and the driver handling characteristics can be characterized by these parameters [32]. The basic driver model is considered to be a proportional and differential controller with a delay element and attempts to minimize the difference between the vehicle trajectory and the desired trajectory. The applicable steering wheel angle for representing the driver’s steering characteristics is described as follows [32]:

(9) $$\delta_{h}^{i}=\frac{G_{h}^{i}(1+\tau_{h}^{i}s)}{1+T_{h}^{i}s}(Y_{des}^{i}-Y^{i})$$

where $Y_{des}^{i}$ and $Y^{i}$ are the target and current lateral positions of the vehicle’s center of gravity, $G_{h}^{i}$ is the steering proportional gain, $\tau_{h}^{i}$ and $T_{h}^{i}$ are derivative time constant and response time delay, respectively, and s is the Laplace operator. By assuming that the gear ratio of the steering system is $R_{g}$, $\delta_{f}^{i}=R_{g}\delta_{h}^{i}$, and the driver model in Equation (9) can be rewritten in the form of differential equation:

(10) $${\dot{\delta }}_{f}^{i}=-\frac{1}{T_{h}^{i}}\delta_{f}^{i}+\frac{R_{g}G_{h}^{i}}{T_{h}^{i}}(Y_{des}^{i}-Y^{i})+\frac{R_{g}G_{h}^{i}\tau_{h}^{i}}{T_{h}^{i}}({\dot{Y}}_{des}^{i}-{\dot{Y}}^{i})$$

**Figure 2:**
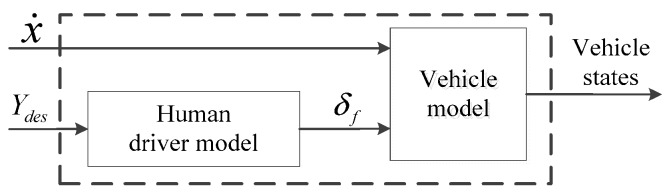
Structure of the driver-vehicle system.

The systems described in Equations (1)–(8) and (10) can be assembled as a driver-vehicle system, as shown in Figure 2. By combining the two systems and the V2V information interactive model, the information interaction system is formed, and expressed as follows:

(11) $$\dot{x}\left(t\right)=f\left(x\left(t\right),u\left(t\right)\right)$$

where the state variables of this system consist of the states of the two vehicles, defined as follows:

(12) $$x={\left[{\dot{y}}^{p},{\dot{x}}^{p},\varphi^{p},{\dot{\varphi }}^{p},Y^{p},X^{p},\delta_{f}^{p},{\dot{y}}^{q},{\dot{x}}^{q},\varphi^{q},{\dot{\varphi }}^{q},Y^{q},X^{q},\delta_{f}^{q}\right]}^{T}$$

The control variable of the system is the front wheel steering angle of two vehicles:

(13) $$u=\delta_{f}^{p}$$

### 2.3. Predictive Model of Moving Obstacle Trajectory

In the actual environment, since the external environment is dynamic, the trajectory tracking control under the given trajectory does not guarantee the autonomous vehicle to deal with any problem accurately. The obstacle avoidance function which obstacles are fixed or trajectories are known has been unable to meet the requirements of dynamic obstacle avoidance. In other words, the vehicle state information at the moment k is shared by two vehicles and the vehicle p performs the obstacle avoidance function according to the information at the moment k. Since the state information of the vehicle q at the moment k+1 cannot be obtained in advance, there is still a collision risk between vehicles at the moment k+1. It is necessary to predict the trajectory by using the state information at the moment k provided by other vehicles to realize the obstacle avoidance function.

Based on simplified driver model, derived from the equation [33]:

(14) $$\frac{d\beta_{pre}^{q}}{dt}=-\frac{2(k_{1}+k_{2})}{m{\dot{x}}^{q}}\beta_{pre}^{q}+\frac{2k_{1}}{m{\dot{x}}^{q}}\delta^{q}-[1+\frac{2(l_{f}k_{1}-l_{r}k_{2})}{m{\left({\dot{x}}^{q}\right)}^{2}}]{\dot{\varphi }}_{pre}^{q}$$

(15) $$\frac{d{\dot{\varphi }}_{pre}^{q}}{dt}=-\frac{2(l_{f}k_{1}-l_{r}k_{2})}{I}\beta_{pre}^{q}+\frac{2l_{f}k_{1}}{I}\delta^{q}-\frac{2(l_{f}^{2}k_{1}-l_{r}^{2}k_{2})}{I{\dot{x}}^{q}}\varphi_{pre}^{q}$$

Based on the front wheel steering angle $\delta^{q}$ of the interactive information, the vehicle sideslip angle $\beta_{pre}^{q}$ and the yaw angle $\varphi_{pre}^{q}$ can be estimated and compared with the vehicle sideslip angle $\beta^{q}$ and the yaw angle $\varphi^{q}$ in the real-time interactive information.

(16) $$\frac{dX_{pre}^{q}}{dt}={\dot{x}}^{q}\cos(\beta_{pre}^{q}+\varphi_{pre}^{q})$$

(17) $$\frac{dY_{pre}^{q}}{dt}={\dot{x}}^{q}\sin(\beta_{pre}^{q}+\varphi_{pre}^{q})$$

The above equation is integrated to obtain the vehicle center of mass position:

(18) $$X_{pre}^{q}=X_{0}^{q}+{\dot{x}}^{q}\int_{0}^{t}\cos(\beta_{pre}^{q}+\varphi_{pre}^{q})dt$$

(19) $$Y_{pre}^{q}=Y_{0}^{q}+{\dot{x}}^{q}\int_{0}^{t}\sin(\beta_{pre}^{q}+\varphi_{pre}^{q})dt$$

The first derivative of the trajectory curve indicates the value of the front wheel steering angle $\delta_{pre}^{q}$ of the vehicle while the direction of the steering angle is determined by the concavity and convexity of the trajectory curve. Thus, the trajectory of the vehicle q at time k+1 is predicted based on the information at time k. The prediction model is validated by two typical conditions: (1) vehicle q with a constant longitudinal velocity of 15 m/s; (2) vehicle q with a variable longitudinal velocity from 13 m/s to 20 m/s, and the longitudinal acceleration is 1 m/s^2^.

**Figure 3:**
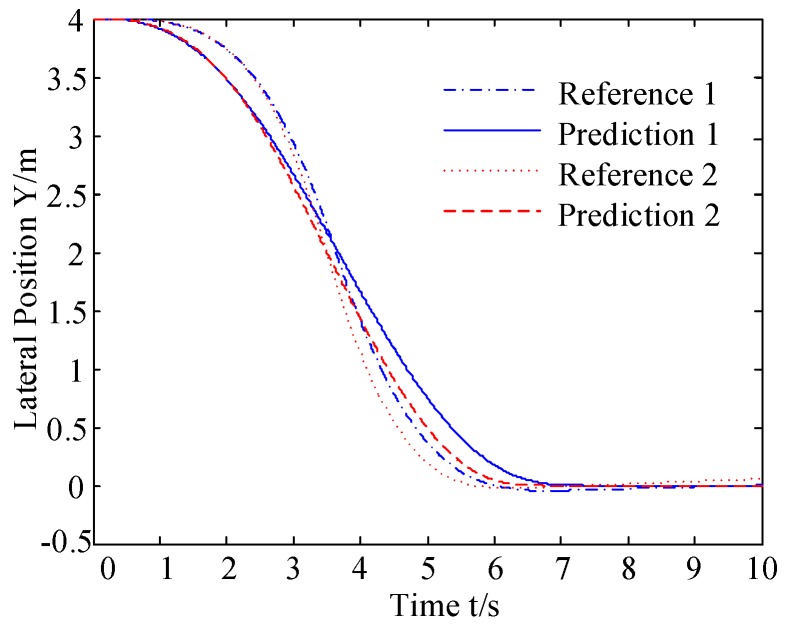
Comparison of reference trajectories and predicted trajectories.

**Figure 4:**
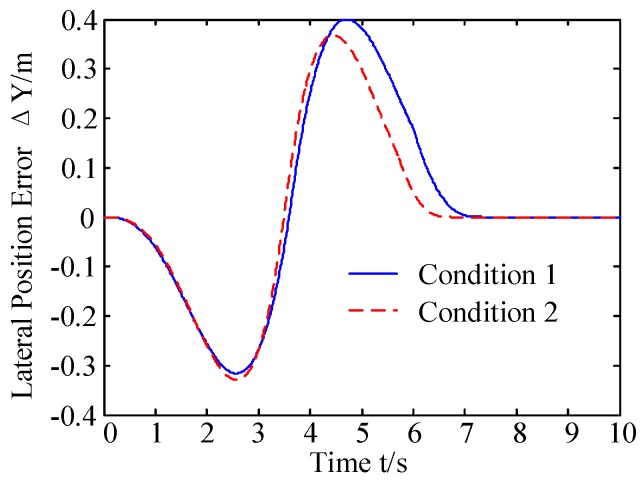
Error of prediction trajectory and reference trajectory of obstacle.

The reference trajectory and the predicted trajectory are compared in Figure 3. The error between the predicted trajectory and the reference trajectory is shown in Figure 4.

As shown in Figure 3 and Figure 4, in two conditions, the prediction trajectory is generated by the obstacle trajectory prediction model, and the error between predicted trajectory and reference trajectory is small. As shown in Figure 4, before the two lane change process, the error between predicted trajectory and reference trajectory is negative, making the predicted trajectory closer to the autonomous vehicle, enabling the autonomous vehicle to perceive obstacles ahead and achieve collision avoidance control. After the crossing, the error value is positive which helps the autonomous vehicle make a more rapid decision on whether there is a threat or not.

## 3. Controller Design

**Figure 5:**
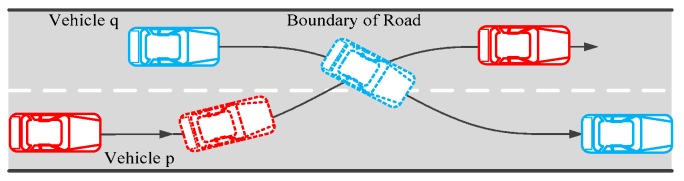
Typical lane changing scenario.

In this paper, a typical vehicle-to-vehicle (V2V) encountering scenario of lane exchanging with two vehicles maneuvered to exchange lanes is built. That is, two vehicles traveling on contiguous lanes in the same direction are maneuvered to exchange lanes, as shown in Figure 5. The controller is designed for this conflicting scenario.

**Figure 6:**
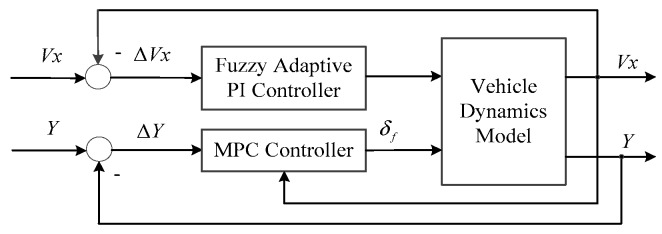
Vehicle control framework.

Vehicle tracking control requirements are mainly divided into longitudinal velocity control and lateral position control. The control framework is shown as Figure 6.

The integrated information interaction model constructs two vehicle systems into a V2V information exchange system. This section first introduces the overall control framework, and then gives the longitudinal control of the fuzzy adaptive PI controller design and lateral control design of the LTV MPC controller design.

### 3.1. Control Framework

**Figure 7:**
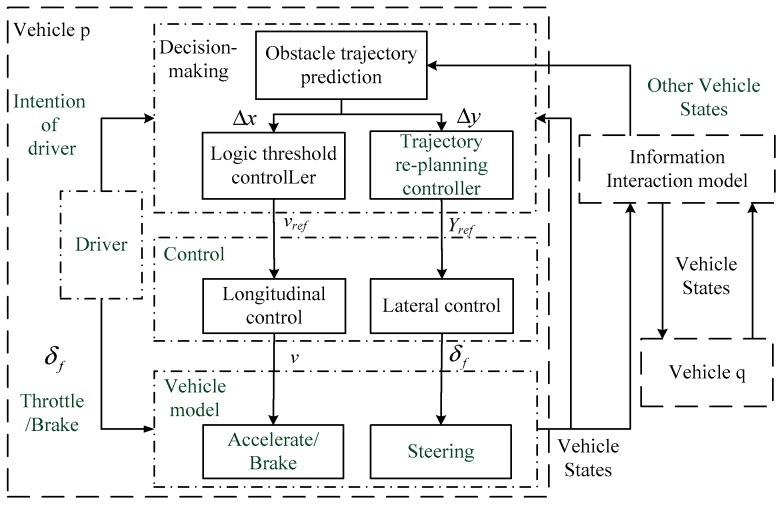
Lane-exchanging vehicle trajectory re-planning control framework.

The trajectory re-planning control framework for lane exchanging of two vehicles is described in Figure 7, where intentions of the drivers can be given by the front tire steering angles and throttle/brake inputs and feedback to the trajectory re-planning controller. Different drivers have different handling characteristics for the vehicle. The driver’s character, age, and other factors have a great impact on the handling of the vehicle. The data of the driver’s handling characteristics are available in the onboard controllers. Communicate through the information interactive model aiming to make the state information between the two vehicles can be interactive. The desired velocity and desired trajectory determined by the controller are tracked by the longitudinal and lateral controllers, respectively.

In the lane changing process, there are conflicting threats to the desired trajectory of the vehicles. If two vehicles do not take proper measures, collisions are inevitable. Before lane exchanging, changing the vehicle velocity or changing the desired trajectory of the vehicle is an effective way of avoiding collisions. In this paper, the MPC controller is proposed to re-plan the optimal desired trajectory to avoid obstacles. The focus of this paper is trajectory re-planning function in vehicle lane exchanging process. The control framework in Figure 7 adopts fuzzy adaptive PI control and model predictive control to realize the tracking and control of the vehicle’s longitudinal velocity and the desired value of the front wheel steering angle. The information of vehicles is shared by the information interaction model.

### 3.2. Longitudinal Control

Longitudinal control is an important part of autonomous vehicles, and it is one of the most basic and key technologies for autonomous vehicles. The longitudinal controller is designed and analyzed in this section to enable the accurate velocity tracking. The longitudinal control of autonomous vehicles is influenced by external factors. The controlled object has strong non-linearity, and it is difficult to meet the requirements of rapid and accurate tracking reference velocity with the traditional control method. The adaptive control can adjust the structure (parameters) of the controller in real time to ensure that the system always running in the ideal state and the fuzzy adaptive PI control method is adopted in this paper. The fuzzy control rule developed in the fuzzy PI control method is a reflection of the driver’s knowledge and experience and the driver’s handling behavior is taken into account during the design of the controller [34].

#### 3.2.1. PI Controller Design

The continuous proportional-integral-derivative (PID) control algorithm cannot be directly used as a controller and needs to be discretized. The positional PID control algorithm is expressed as follows [35]:

(20) $$\begin{array}{cc} u\left(k\right) & =k_{p}\left(error\left(k\right)+\frac{T}{T_{I}}\sum_{j=0}^{k}error\left(j\right)+\frac{T_{D}}{T}\left(error\left(k\right)-error\left(k-1\right)\right)\right)\\ & =k_{p}error\left(k\right)+k_{i}\sum_{j=0}^{k}error\left(j\right)+k_{d}\left(error\left(k\right)-error\left(k-1\right)\right) \end{array}$$

where $k_{i}=k_{p}*T/T_{I}$, $k_{d}=k_{p}T_{D}/T$. T, $T_{I}$, and $T_{D}$ are the sampling time, integration time, derivative time, respectively. k=1,2,⋯ is the sampling number, error(k−1) and error(k−1) are the deviation signals obtained at the time of k−1 and k. u(k) in Equation (20) is the output of the positional PID control algorithm. Set $k_{d}=0$. According to the recursive principle:

(21) $$u\left(k-1\right)=k_{p}\left(error\left(k-1\right)+k_{i}\sum_{j=0}^{k-1}error\left(j\right)\right)$$

Equation (22) is subtracted by Equations (20) and (21):

(22) $$\Delta u\left(k\right)=k_{p}\left(error\left(k\right)-error\left(k-1\right)\right)+k_{i}error\left(k\right)$$

Δu(k) is the output of the incremental PI control algorithm. The control variable u(k) is obtained by Equation (23):

(23) u(k)=u(k−1)+Δu(k)

#### 3.2.2. Fuzzy Adaptive PI Controller Design

**Figure 8:**
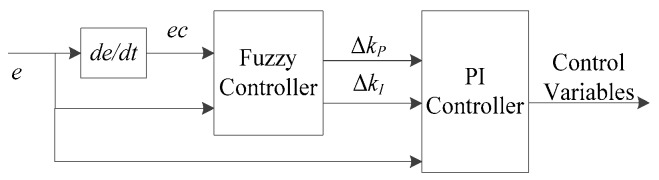
Structure of fuzzy adaptive PI controller.

Aiming at the velocity tracking problem of autonomous vehicles in this paper, the deviation between the reference velocity and the actual velocity of the vehicle e and the rate of deviation change ec are taken as input. The outputs of the fuzzy controller $\Delta k_{P},\Delta k_{I}$ are the increment of $k_{P},k_{I}$ for adjusting the two important parameters of the PI controller. The structure of the fuzzy adaptive PI controller is shown in Figure 8.

The input variable e,ec and the output variable $\Delta k_{P},\Delta k_{I}$ are all on the fuzzy set {−1,0,1}. The quantization factors were 1/4 and 1/10 respectively. The scale factor is 50 and 0.1. Using the fuzzy language name {N,Z,P}, representing negative, zero, positive. The fuzzy control rules are shown in Table 1 and Table 2.

(1) The setting principle of $k_{P}$: When the response is in the ascending process (e is P), $\Delta k_{P}$ takes a positive value to increase $k_{P}$. When overshoot (e is N), $\Delta k_{P}$ takes a negative value to decrease $k_{P}$. When the error is near zero (e is Z), there are three cases: when ec is N, the overshoot is getting bigger and bigger, then $\Delta k_{P}$ takes negative; when ec is Z, in order to reduce the error, $\Delta k_{P}$ is positive; when ec is P, the positive error is getting bigger, then $\Delta k_{P}$ is positive. The fuzzy control rules of $k_{P}$ are shown in Table 1.

(2) The setting principle of $k_{I}$: using the integral separation strategy, when the error value is near zero, $\Delta k_{I}$ is positive, otherwise zero. The fuzzy control rules of $k_{I}$ is shown in Table 2.

**Figure 9:**
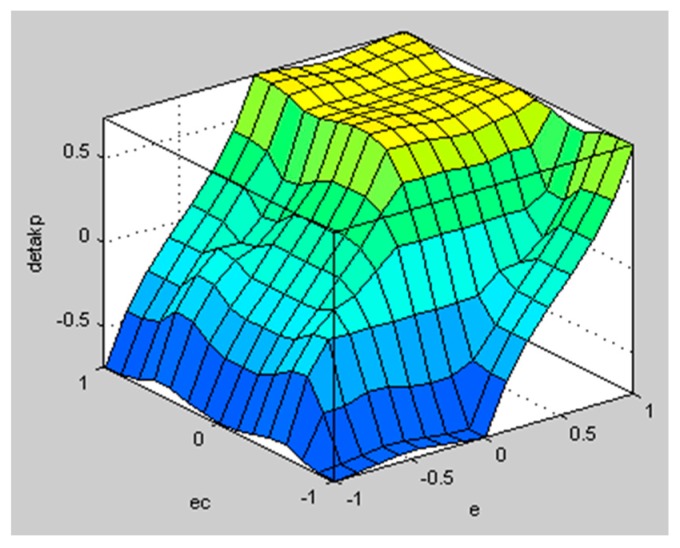
Fuzzy surface of $\Delta k_{P}$.

**Figure 10:**
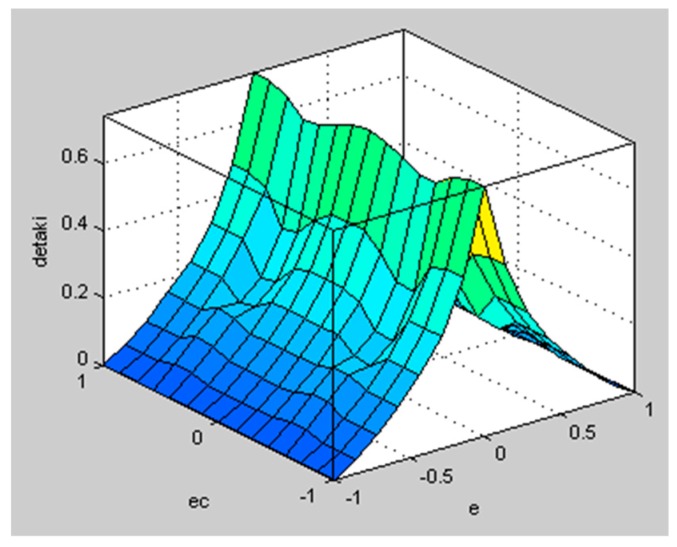
Fuzzy surface of $\Delta k_{I}$.

The well-known Mamdani inference method is used to solve the fuzzy implication. The fuzzy surfaces are shown in Figure 9 and Figure 10:

### 3.3. Lateral Control

This paper focuses on the study of the lateral obstacle avoidance control of autonomous vehicles. The longitudinal velocity changes when necessary. The lateral controller of the autonomous vehicle is based on the planned path. The non-linear MPC have been developed for vehicle lateral control. The nonlinear MPC requires solving the optimal control problem on the finite prediction step. Although these problems are convex in the linear MPC, they are no longer convex in the nonlinear MPC. This poses a challenge to nonlinear MPC stability theory and numerical solutions, and does not satisfy real-time constraints [36]. In this paper, a linear model predictive control method is proposed to design the trajectory tracking controller.

#### 3.3.1. Discretization and Linearization

At the moment of t, the nonlinear vehicle dynamics model $\left(x_{0}\left(t\right),u_{0}\left(t-1\right)\right)$ was developed for Taylor [37]:

(24) $$\dot{x}\left(t\right)=f\left(x_{0}\left(t\right),u_{0}\left(t-1\right)\right)+{\frac{\partial f}{\partial x}|}_{x_{0}\left(t\right),u_{0}\left(t-1\right)}\left(x\left(t\right)-x_{0}\left(t\right)\right)+{\frac{\partial f}{\partial u}|}_{x_{0}\left(t\right),u_{0}\left(t-1\right)}\left(u\left(t\right)-u_{0}\left(t-1\right)\right)$$

where: ${\frac{\partial f}{\partial x}|}_{x_{0}\left(t\right),u_{0}\left(t-1\right)}$, ${\frac{\partial f}{\partial u}|}_{x_{0}\left(t\right),u_{0}\left(t-1\right)}$ are calculated by the following equation [37]:

$$\begin{array}{l} {\frac{\partial f}{\partial x}|}_{x_{0}\left(t\right),u_{0}\left(t-1\right)}=[\begin{array}{ccccccc} \frac{-2(C_{cf}+C_{cr})}{m{\dot{x}}^{i}} & -{\dot{\varphi }}^{i} & 0 & -{\dot{x}}^{i}+\frac{2(C_{cr}l_{r}-C_{cf}l_{f})}{m{\dot{x}}^{i}} & 0 & 0 & \frac{2C_{cf}}{m}\\ {\dot{\varphi }}^{i} & 0 & 0 & {\dot{y}}^{i} & 0 & 0 & 0\\ 0 & 0 & 0 & 1 & 0 & 0 & 0\\ \frac{2(C_{cr}l_{r}-C_{cf}l_{f})}{I_{Z}{\dot{x}}^{i}} & 0 & 0 & -\frac{2(C_{cr}l_{r}^{2}+C_{cf}l_{f}^{2})}{I_{Z}{\dot{x}}^{i}} & 0 & 0 & \frac{2C_{cf}l_{f}}{I_{Z}}\\ 1 & 0 & {\dot{x}}^{i} & 0 & 0 & 0 & 0\\ 0 & 1 & -{\dot{y}}^{i} & 0 & 0 & 0 & 0\\ \frac{-R_{g}G_{h}^{i}\tau_{h}^{i}}{T_{h}^{i}} & 0 & \frac{-R_{g}G_{h}^{i}\tau_{h}^{i}}{T_{h}^{i}}{\dot{x}}^{i} & 0 & \frac{-R_{g}G_{h}^{i}}{T_{h}^{i}} & 0 & \frac{-1}{T_{h}^{i}} \end{array}],\\ {\frac{\partial f}{\partial u}|}_{x_{0}\left(t\right),u_{0}\left(t-1\right)}={\left[\begin{array}{ccccccc} \frac{2C_{cf}}{m} & 0 & 0 & \frac{2C_{cf}l_{f}}{I_{Z}} & 0 & 0 & -\frac{1}{T_{h}^{i}} \end{array}\right]}^{T} \end{array}$$

make the following settings:

$$\hat{u}=u_{0}\left(t-1\right)\;\;\;\;\;\hat{x}=x_{0}\left(t\right)$$

Then Equation (24) can be expressed as:

(25) $$\dot{x}\left(t\right)=\dot{\hat{x}}+{\frac{\partial f}{\partial x}|}_{x_{0}\left(t\right),u_{0}\left(t-1\right)}\left(x\left(t\right)-\hat{x}\right)+{\frac{\partial f}{\partial u}|}_{x_{0}\left(t\right),u_{0}\left(t-1\right)}\left(u\left(t\right)-\hat{u}\right)$$

To be discretized [37]:

(26) $$x\left(k+1\right)=\hat{x}\left(k+1\right)+A\left(x\left(k\right)-\hat{x}\left(k\right)\right)+B\left(u\left(k\right)-\hat{u}\left(k\right)\right)$$

Equation (26) is organized into a linear discretization of vehicle dynamics model of the equation of state:

(27) x(k+1)=Ax(k)+Bu(k)+d(k)

where $d\left(k\right)=\hat{x}\left(k+1\right)-A\hat{x}\left(k\right)-B\hat{u}\left(k\right)$. Since the two vehicle state information needs to be processed simultaneously by the MPC controller,

we define $A=diag(\begin{array}{cc} A^{p} & A^{q} \end{array})$, where $A^{i}\left(i=p,q\right)=I+T\cdot {\frac{\partial f}{\partial x}|}_{x_{0}\left(t\right),u_{0}\left(t-1\right)}$, T is sampling time,
we define $B=diag(\begin{array}{cc} B^{p} & B^{q} \end{array})$, where $B^{i}\left(i=p,q\right)=T\cdot {\frac{\partial f}{\partial u}|}_{x_{0}\left(t\right),u_{0}\left(t-1\right)}$, T is sampling time.

Change the input of Equation (27) from control variable u(k) to control increment Δu(k) to obtain the state equation of vehicle dynamics model as shown in Equation (28):

(28) $$\tilde{x}\left(k+1\right)=\tilde{A}\tilde{x}\left(k\right)+\tilde{B}\Delta u\left(k\right)+\tilde{d}\left(k\right)$$

where $\tilde{x}\left(k\right)=\left[\begin{array}{c} x\left(k\right)\\ u\left(k-1\right) \end{array}\right]$, $\tilde{A}=\left[\begin{array}{cc} A_{n\times n} & B_{n\times m}\\ 0_{m\times n} & I_{m\times m} \end{array}\right]$, $\tilde{B}=\left[\begin{array}{c} B_{n\times m}\\ I_{m\times m} \end{array}\right]$, Δu(k)=u(k)−u(k−1), $\tilde{d}\left(k\right)=\left[\begin{array}{c} d\left(k\right)\\ 0 \end{array}\right]$, m and n are the dimension of the control variables and state variables, respectively.

In this paper, the yaw angle $\varphi^{i}$ and the longitudinal position of the vehicle $Y^{i}$ are selected as the output of the state space. In order to integrate the state information of two vehicles into the MPC controller and maintain the dimension uniformity, the output is:

(29) $$\tilde{y}\left(k\right)=\tilde{C}\tilde{x}\left(k\right)$$

where $\tilde{y}\left(k\right)={\left[\varphi^{p},Y^{p},\varphi^{q},Y^{q}\right]}^{T}$, $\tilde{C}=\left[\begin{array}{cc} \Phi & 0\\ 0 & \Phi \end{array}\right]$, $\Phi =\left[\begin{array}{cccccccc} 0 & 0 & 1 & 0 & 0 & 0 & 0 & 0\\ 0 & 0 & 0 & 0 & 1 & 0 & 0 & 0 \end{array}\right]$.

#### 3.3.2. Obstacle Avoidance Function

In the actual environment, since the external environment is dynamic, the trajectory tracking control under the given trajectory does not guarantee the autonomous vehicle to deal with any problem accurately. When there is an obstacle in a given desired trajectory, the autonomous vehicle re-plans the trajectory according to the information of the obstacle to avoid the obstacle and then continues to track the desired trajectory.

In this paper, based on the trajectory tracking control, the MPC-based local planning layer is proposed to form a new control system. The local path planning controller re-plans the local desired trajectory according to the reference path information and the obstacle information obtained by the sensor. At the same time, the local desired trajectory information is input to the tracking control layer, thus the global reference path is tracked while the obstacle avoidance is realized.

The basic idea of the penalty function is to adjust the size of the function value according to the distance deviation between the obstacle and the target, and the closer the distance is, the larger the function value is. The penalty function is as follows:

(30) $$J_{obs,i}=\frac{S_{obs}(v_{x}^{2}+v_{y}^{2})}{{\left(x_{i}-x_{0}\right)}^{2}+{\left(y_{i}-y_{0}\right)}^{2}+\Delta }$$

where $S_{obs}$ is the weight coefficient, $\left(x_{i},y_{i}\right)$ is the position of the obstacle in the body coordinate system, $\left(x_{0},y_{0}\right)$ is the position of the center of mass of the vehicle, Δ is a small positive number, preventing the denominator from being zero. The target of local trajectory planning is to achieve the obstacle avoidance function while minimizing the deviation from the global reference path. The local trajectory re-planning model predictive controller is as follows:

(31) $$\begin{array}{c} \underset{U_{t}}{\min}\sum_{i=1}^{N_{p}}{\left\|\tilde{y}(k+i|k)-y_{ref}(k+i|k)\right\|}_{S}^{2}+{\left\|U_{i}\right\|}_{R}^{2}+J_{obs,i}\\ s.t.U_{\min}\leq U_{t}\leq U_{\max} \end{array}$$

where $\tilde{y}(k+i|k)$ and $y_{ref}(k+i|k)$ are the output value and desired value at the moment k+i. $U_{i}$ is the control variable sequence.

**Figure 11:**
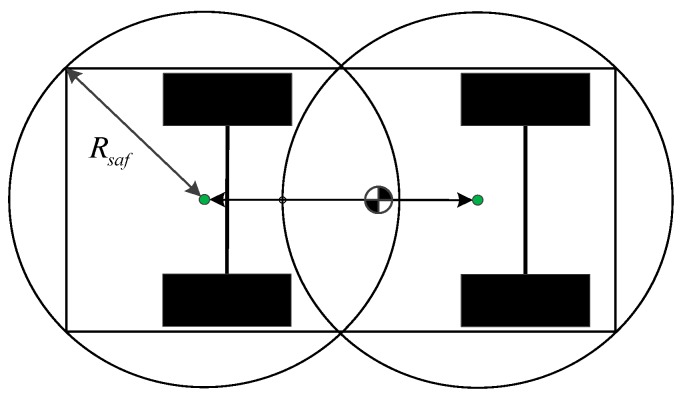
Circular expansion of the vehicle shape.

In order to make the result of trajectory planning feasible, the expansion of the obstacle is carried out according to the vehicle body size. In this paper, circles with the same radius are used instead of the vehicle body rectangle, as shown in Figure 11. In order to ensure safe driving, the distance between the vehicle body and the center of the obstacle must be greater than the radius of any circle.

In [38], a trajectory planning algorithm based on polynomial fitting is proposed, which uses a simplified spherical extension polyhedron to express the obstacle and add dynamic constraints and it is easier to generate smooth trajectories by selecting the coefficients to select the permissible trajectories. The fifth order polynomial is used as the fitting curve:

(32) $$\begin{array}{l} Y=a_{0}t^{5}+a_{1}t^{4}+a_{2}t^{3}+a_{3}t^{2}+a_{4}t^{1}+a_{5}\\ \varphi =b_{0}t^{5}+b_{1}t^{4}+b_{2}t^{3}+b_{3}t^{2}+b_{4}t^{1}+b_{5} \end{array}$$

where $a_{p}=\left[a_{0},a_{1},a_{2},a_{3},a_{4},a_{5}\right],\;b_{p}=\left[b_{0},b_{1},b_{2},b_{3},b_{4},b_{5}\right]$ are the parameters to be solved.

#### 3.3.3. MPC Controller Design

**Figure 12:**
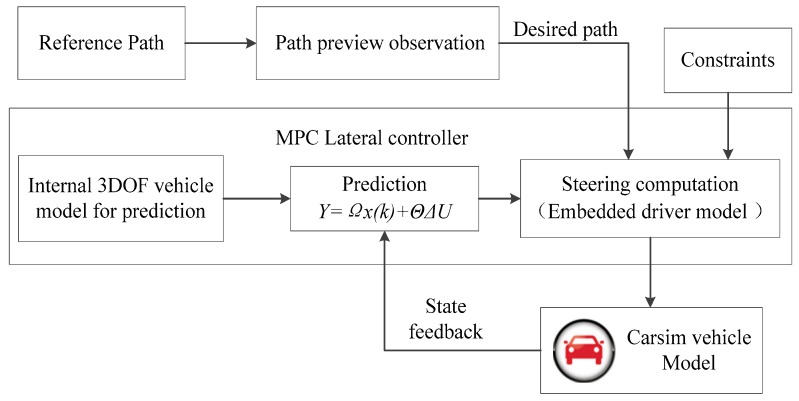
The model structure of the model predictive control (MPC) lateral controller.

The structure of the model predictive controller is shown in Figure 12.

The MPC controller’s task is to solve the optimal solution of the trajectory re-planning under the premise of satisfying the various constraints such as driver’s operation, obstacle avoidance, and the mechanical structure of the vehicle. The vehicle information interaction system includes the states of two connected vehicles. The states of the model are:

$$x={\left[{\dot{y}}^{p},{\dot{x}}^{p},\varphi^{p},{\dot{\varphi }}^{p},Y^{p},X^{p},\delta_{f}^{p},{\dot{y}}^{q},{\dot{x}}^{q},\varphi^{q},{\dot{\varphi }}^{q},Y^{q},X^{q},\delta_{f}^{q}\right]}^{T}$$

The control variable is the front wheel steering angle $u=\delta_{f}^{p}$.

The prediction horizon of the system is defined as $N_{p}$ and control horizon is defined as $N_{c}$. At time k, the output of the vehicle system at time $k+1,\cdots ,k+N_{p}$ is predicted:

(33) $$Y_{out}\left(k\right)=\Omega \left(k\right)\tilde{x}\left(k\right)+\Theta \left(k\right)\Delta U\left(k\right)$$

with $Y_{out}\left(k\right)=\left[\begin{array}{c} \tilde{y}\left(k+1|k\right)\\ \tilde{y}\left(k+2|k\right)\\ \vdots \\ \tilde{y}\left(k+N_{p}|k\right) \end{array}\right]$, $\Omega \left(k\right)=\left[\begin{array}{c} \tilde{C}\tilde{A}\\ \tilde{C}{\tilde{A}}^{2}\\ \vdots \\ \tilde{C}{\tilde{A}}^{N_{p}} \end{array}\right]$, $\Theta \left(k\right)=\left[\begin{array}{ccc} \tilde{C}\tilde{B} & 0 & 0\\ \vdots & \vdots & 0\\ \tilde{C}{\tilde{A}}^{N_{c}-1}\tilde{B} & \cdots & \tilde{C}\tilde{B}\\ \vdots & & \vdots \\ \tilde{C}{\tilde{A}}^{N_{p}-1}\tilde{B} & \cdots & \tilde{C}{\tilde{A}}^{N_{p}-N_{c}}\tilde{B} \end{array}\right]$, $\Delta U\left(k\right)=\left[\begin{array}{c} \Delta u\left(k|k\right)\\ \Delta u\left(k+1|k\right)\\ \vdots \\ \Delta u\left(k+N_{c}-1|k\right) \end{array}\right]$.

The optimization problem to be solved in the receding horizon is defined as follows:

(34a) $$\underset{\Delta U_{t,}\varepsilon }{\min}(\sum_{i=1}^{N_{p}}{\left\|\tilde{y}(k+i|k)-y_{ref,local}(k+i|k)\right\|}_{S}^{2}+\sum_{i=0}^{N_{c}-1}{\left\|\Delta u(k+i|k)\right\|}_{R}^{2}+\rho \varepsilon^{2})$$

(34b) $$s.t.\Delta U_{\min}\leq \Delta U_{t}\leq \Delta U_{\max}$$

(34c) $$U_{\min}\leq A\Delta U_{t}+U_{t}\leq U_{\max}$$

(34d) $$y_{hc,\min}\leq y_{hc}\leq y_{hc,\max}$$

(34e) $$y_{sc,\min}-\varepsilon \leq y_{sc}\leq y_{hc,\max}+\varepsilon$$

(34f) ε>0

In Equation (34a), $\tilde{y}(k+i|k)$ and $y_{ref,local}(k+i|k)$ are the output value and desired value of the output at the future moment k+i. $y_{ref,local}=[Y_{ref,local},\varphi_{ref,local}]$ is the local reference trajectory. Δu(k+i|k) is the control variable sequence of future times. ρ is the weighting factor. ε is the relaxation factor. $y_{sc}$ and $y_{hc}$ are soft constraints and hard constraints, respectively.

Since the control increment in the control time domain is unknown. The objective function is designed using the control increment of the vehicle system as a variable. The optimal control increment sequence in the control horizon is obtained by minimizing the optimization of the objective function. In order to ensure the tracking accuracy of the vehicle, the vehicle lateral position deviation and yaw rate deviation are taken into account in the objective function. At the same time, in order to keep the change of the control variables stable and avoid the abrupt change of the control variables, constraints are added to the control increment in the objective function. The relaxation factor is added to ensure that the objective function has a feasible solution.

The state information of the two vehicles is used as the state variable by the controller. In order to maintain dimensions of the matrix unified, the output weight matrix is as follow:

$$S=diag_{N_{p}\times N_{p}}\left(\begin{array}{cccc} s & s & \cdots & s \end{array}\right),$$

where $s=diag_{4\times 4}\left(\begin{array}{cccc} s_{1} & s_{2} & s_{3} & s_{4} \end{array}\right)$,

The control weighting matrix is

$$R=diag_{N_{u}\times N_{c}}\left(\begin{array}{cccc} r & r & \cdots & r \end{array}\right).$$

Equations (34b)–(34f) are the design of the constraint condition. There is a certain limit to the steering range of the front wheel steering angle of the vehicle, therefore, the front wheel steering angle is restrained so as to avoid the fact that the front wheel steering angle is beyond the actual range. The constraint for the control variable is set to:

(35) $$U_{\min}\left(k\right)\leq U\left(k\right)\leq U_{\max}\left(k\right)$$

In order to ensure that the vehicle trajectory tracking process is more stable, the control increment is constrained:

(36) $$\Delta U_{\min}\left(k\right)\leq \Delta U\left(k\right)\leq \Delta U_{\max}\left(k\right)$$

Set relaxation factor:

(37) 0≤ε≤10

By Equations (36) and (37):

(38) $$\xi_{\min}\left(k\right)\leq \xi \left(k\right)\leq \xi_{\max}\left(k\right)$$

where $\xi_{\min}\left(k\right)={\left[\begin{array}{cc} \Delta U_{\min}\left(k\right) & 0 \end{array}\right]}^{T}$, $\xi_{\max}\left(k\right)={\left[\begin{array}{cc} \Delta U_{\max}\left(k\right) & 10 \end{array}\right]}^{T}$.

Equation (38) is the upper and lower bounds of the objective function.

In order to solve the objective function, Equation (34) is transformed into the inequality of ΔU(k). The relationship between the control variables of the vehicle system and the control increment at the moment k is as follows:

(39) $$\left[\begin{array}{c} u\left(k|k\right)\\ u\left(k+1|k\right)\\ \vdots \\ u\left(k+N_{c}-1|k\right) \end{array}\right]=\left[\begin{array}{cccc} 1 & 0 & \cdots & 0\\ \vdots & \ddots & \ddots & \vdots \\ \vdots & & \ddots & 0\\ 1 & \cdots & \cdots & 1 \end{array}\right]\left[\begin{array}{c} \Delta u\left(k|k\right)\\ \Delta u\left(k+1|k\right)\\ \vdots \\ \Delta u\left(k+N_{c}-1|k\right) \end{array}\right]+\left[\begin{array}{c} u\left(k-1|k\right)\\ u\left(k-1|k\right)\\ \vdots \\ u\left(k-1|k\right) \end{array}\right]$$

written in matrix form:

(40) $$U\left(k\right)=G\Delta U\left(k\right)+\tilde{U}$$

The Equation (35) can be converted to:

(41) $$U_{\min}\left(k\right)\leq G\Delta U\left(k\right)+\tilde{U}\leq U_{\max}\left(k\right)$$

Constrain the output of the vehicle system:

(42) $$Y_{out\;\min}\left(k\right)\leq Y_{out}\left(k\right)\leq Y_{out\;\max}\left(k\right)$$

The Equation (42) is expressed as:

(43) $$Y_{out\;\min}\left(k\right)\leq \Omega \left(k\right)\tilde{x}\left(k\right)+\Theta \left(k\right)\Delta U\left(k\right)+\Omega \left(k\right)\tilde{x}\left(k\right)\leq Y_{out\;\max}\left(k\right)$$

According to the dimension of the state information, Equation (44) is sorted by Equations (41) and (43):

(44) $$A^{*}\left(k\right)\xi \left(k\right)\leq b^{*}\left(k\right)$$

with $A^{*}\left(k\right)=\left[\begin{array}{cc} G & 0\\ -G & 0\\ \Theta \left(k\right) & 0\\ -\Theta \left(k\right) & 0 \end{array}\right]$, $b^{i*}\left(k\right)=\left[\begin{array}{c} U_{\max}\left(k\right)-\tilde{U}\\ -U_{\min}\left(k\right)+\tilde{U}\\ Y_{out\;\max}\left(k\right)-\Omega \left(k\right)\tilde{x}\left(k\right)-\Omega \left(k\right)\tilde{x}\left(k\right)\\ -Y_{out\;\min}\left(k\right)+\Omega \left(k\right)\tilde{x}\left(k\right)+\Omega \left(k\right)\tilde{x} \end{array}\right]$,

The problem is transformed into a single objective optimization problem with upper and lower bound constraints and inequality constraints. By optimizing Equation (34), the optimal control increment sequence in the control horizon $N_{c}$ can be obtained as:

(45) $$\Delta U^{*}\left(k\right)={\left[\begin{array}{c} \Delta u^{*}\left(k|k\right)\\ \Delta u^{*}\left(k+1|k\right)\\ \cdots \\ \Delta u^{*}\left(k+N_{c}-1|k\right) \end{array}\right]}^{T}$$

The MPC algorithm does not apply the optimal sequence to the vehicle system one by one, but only the first control increment $\Delta u^{*}\left(k|k\right)$ is applied to the vehicle system. Equation (46) is used to obtain the optimal control at the current moment acting on the vehicle system.

(46) $$u\left(k|k\right)=u\left(k-1|k\right)+\Delta u^{*}\left(k|k\right)$$

The driver model is embedded in the MPC controller using Equation (24). This section chooses young and aged, respectively, as a comparison of driver characteristics. Set $G_{h}=0.8$, $T_{h}=0.13$, $\tau_{h}=1.1$ as a young driver characteristic parameter and $G_{h}=0.5$, $T_{h}=0.18$, $\tau_{h}=1.1$ as an aged driver characteristic parameter.

The parameters of MPC controller are shown in Table 3.

## 4. Simulation Results

In this section, the nonlinear vehicle dynamics model in Carsim software is used and co-simulation is carried out by Simulink. Select the D-Class Sedan model for simulation. The main parameters of the model are shown in Table 4.

We mainly deal with low-traffic scenarios on the roads; thus, make the following conditions and assumptions:

1. Initially, the vehicle q moves in the left lane of the road, then changes lanes on the right lane, ignoring other vehicles and collision threats.
2. When the vehicle is changing lanes, no other vehicle is used as an obstacle to ensure that there are no other conflicts.
3. The vehicle can obtain interactive information accurately including the state information of its own and obstacle vehicle.

There are three simulation scenarios in total for trajectory re-planning controller verification. These include different initial vehicle distances, vehicle constant velocity, and variable velocity. In this paper, the logic threshold switch is designed, and the selection of vehicle velocity and the controller are selected according to the logical relationship between the difference of the longitudinal position and the difference of the lateral position of the vehicles. 

**Figure 13:**
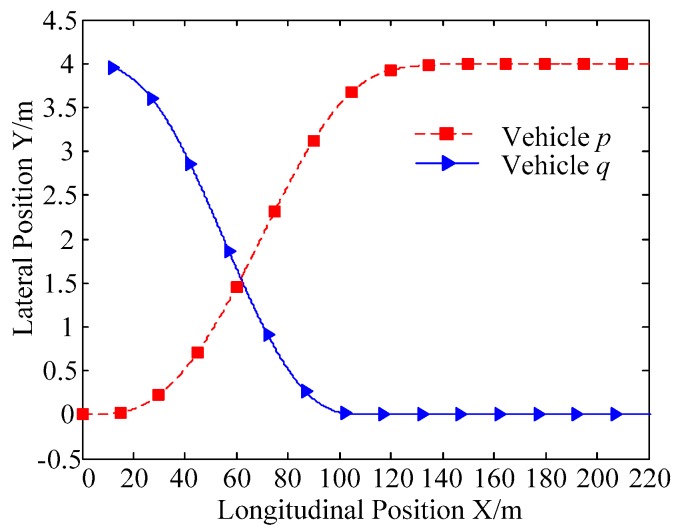
Trajectories of vehicle p and vehicle q.

**Figure 14:**
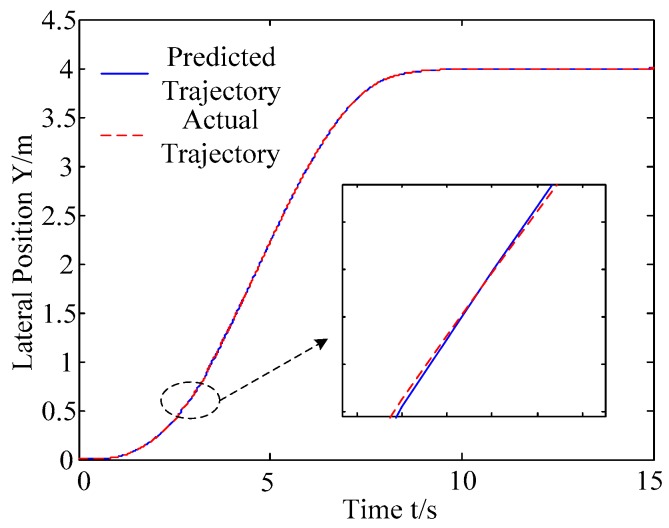
Trajectories with the actual trajectory and the predicted trajectory of the obstacle.

**Figure 15:**
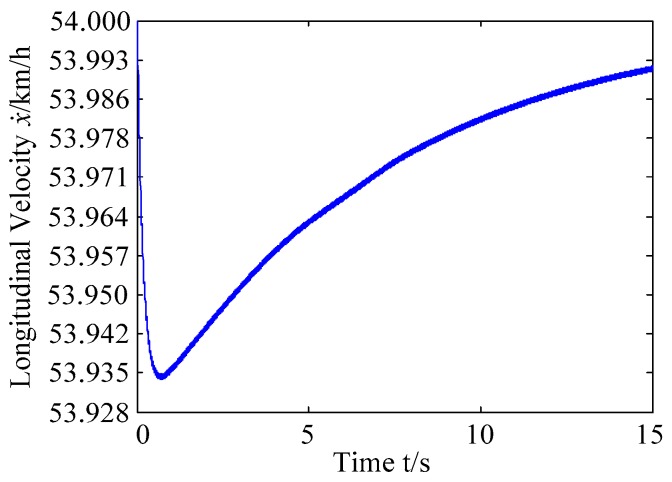
Longitudinal velocity of vehicle p.

**Figure 16:**
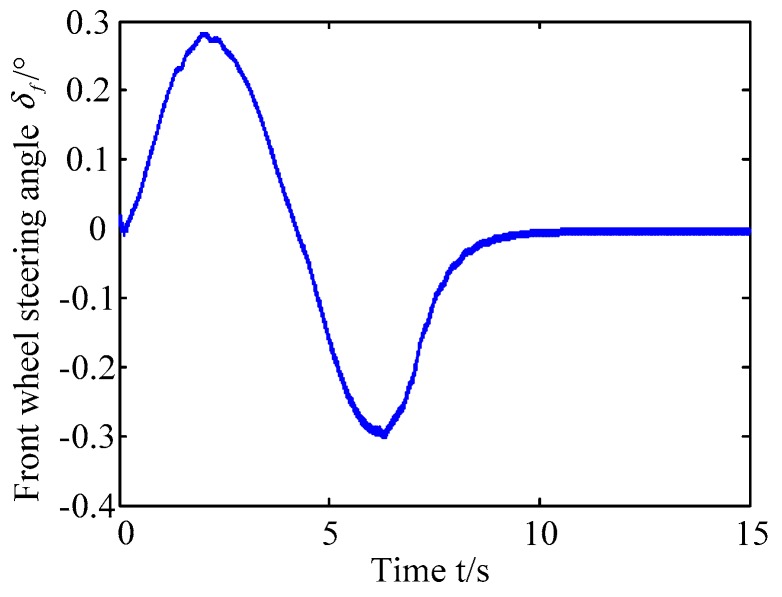
Front wheel steering angle of vehicle p.

**Figure 17:**
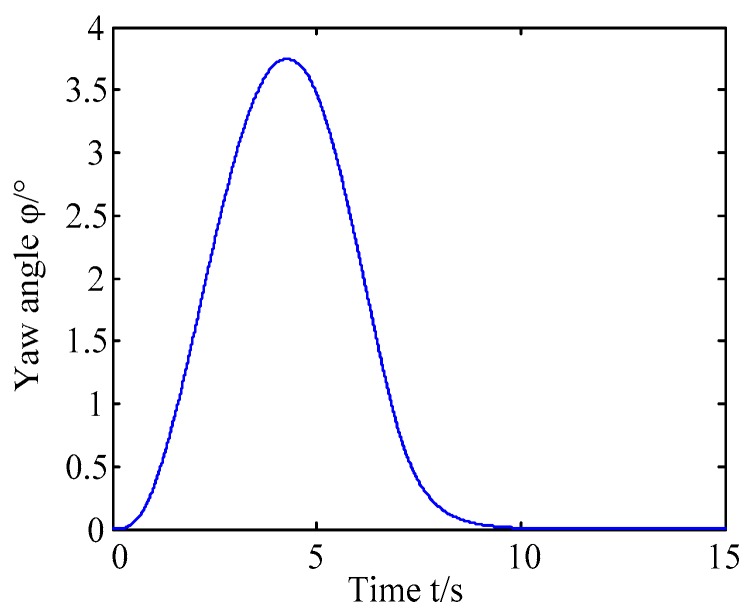
Yaw angle of vehicle p.

**Figure 18:**
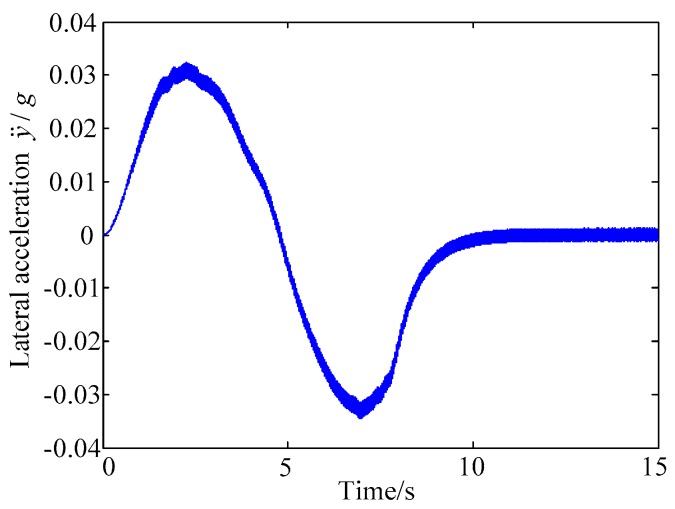
Lateral acceleration of vehicle p.

A. Scenario on larger initial distance. First, we consider a simple two vehicle lane exchanging scenario, as shown in Figure 4. Vehicle p is used as an active vehicle containing a trajectory re-planning controller, and the vehicle q is used as an obstacle vehicle. The initial distance between two vehicles is 12 m. The vehicle q moves at a constant velocity of 54 km/h. The vehicle p has an initial velocity of 54 km/h. The position and trajectories are shown in Figure 13 and Figure 14. Figure 15, Figure 16, Figure 17 and Figure 18 are longitudinal velocity, front wheel steering angle, yaw angle, lateral acceleration of vehicle p, respectively. 

The discrete points of the curve in Figure 13 represent the positions of the vehicle at different times. As shown, there is no collision throughout the time course. The curves in Figure 14 are the dynamic reference trajectories of the controller which are determined by the real-time trajectory and prediction trajectory of obstacle, respectively. The two kinds of dynamic reference trajectories almost coincide, which proves the validity and correctness of the method proposed in this paper. As a result of the large initial distance, the logic threshold controller determines the no conflict between vehicles. Therefore, there is no change in the longitudinal velocity of the vehicle. There is no need to re-plan a new reference trajectory by local trajectory re-planning controller. As shown in Figure 15, although the longitudinal velocity of the vehicle has a very small change, it can be considered that the longitudinal velocity of the vehicle is constant. As shown in Figure 16, Figure 17 and Figure 18, since the local trajectory is not re-planned, the MPC controller is in good condition, and the vehicle is controlled smoothly with good stability.

**Figure 19:**
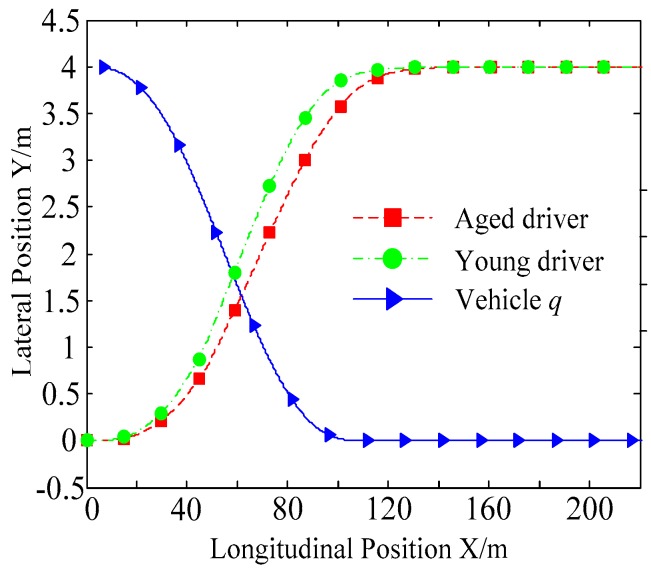
Trajectories of the vehicles driven by different drivers.

**Figure 20:**
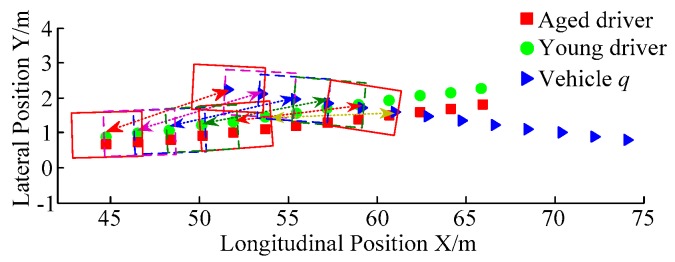
Trajectories of the vehicles driven by different drivers.

**Figure 21:**
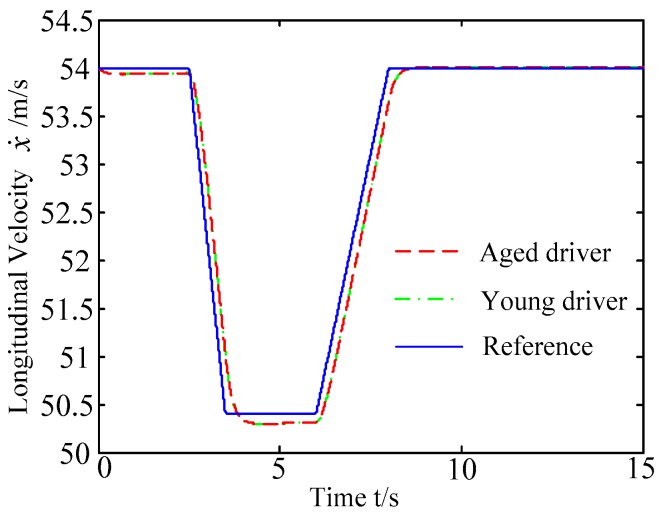
Longitudinal velocities of vehicle p under different handling characteristics.

**Figure 22:**
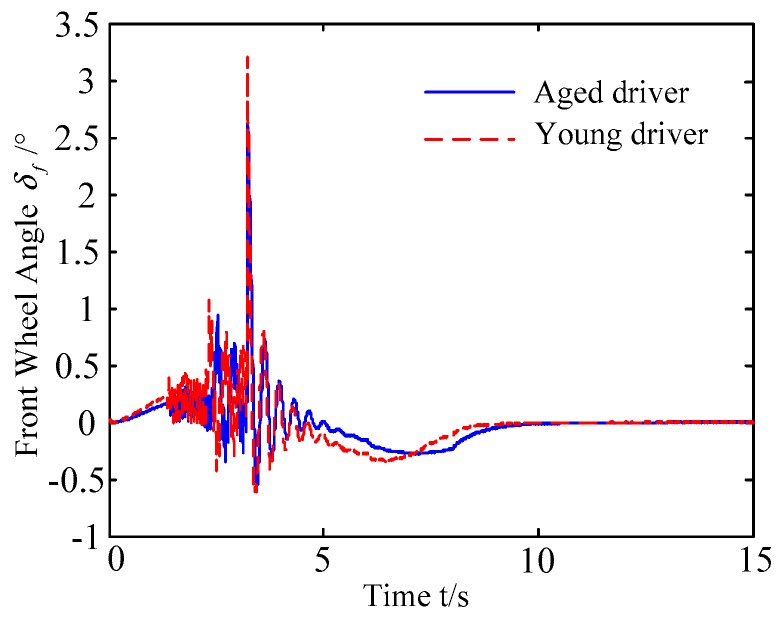
Front wheel angle of vehicle p under different handling characteristics.

**Figure 23:**
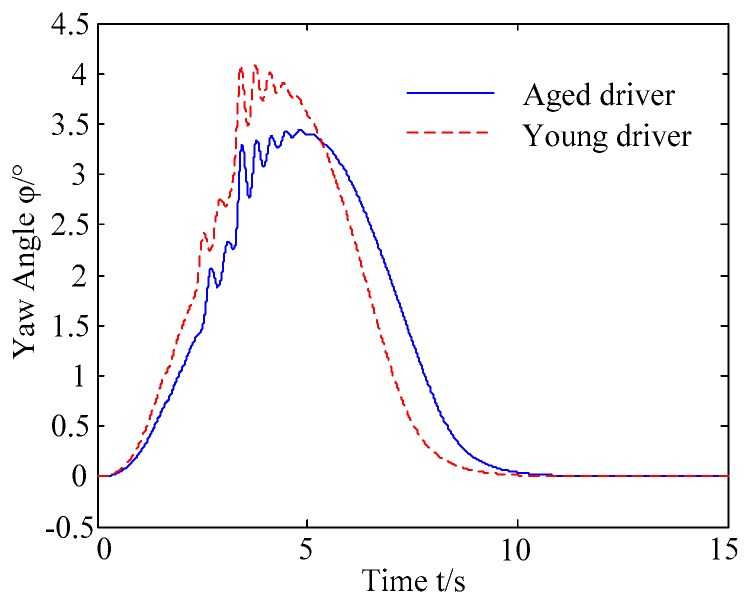
Yaw angle of vehicle p under different handling characteristics.

**Figure 24:**
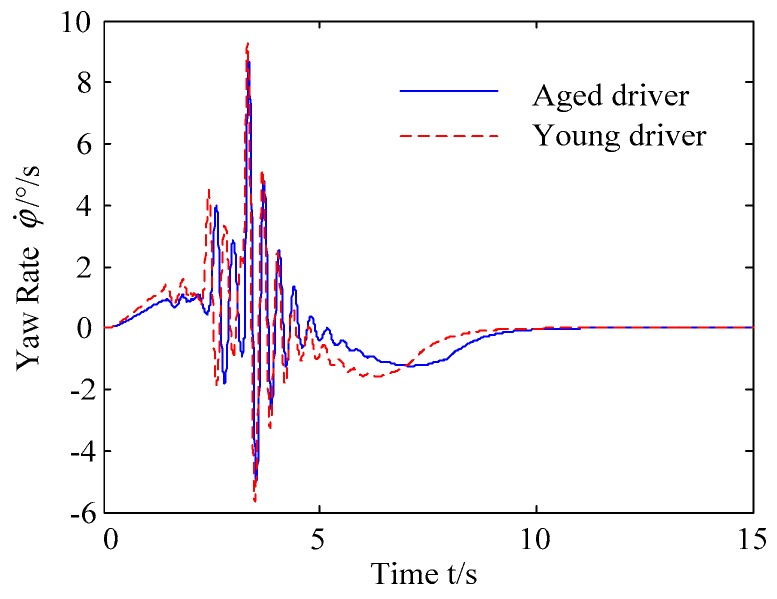
Yaw rate of vehicle p under different handling characteristics.

**Figure 25:**
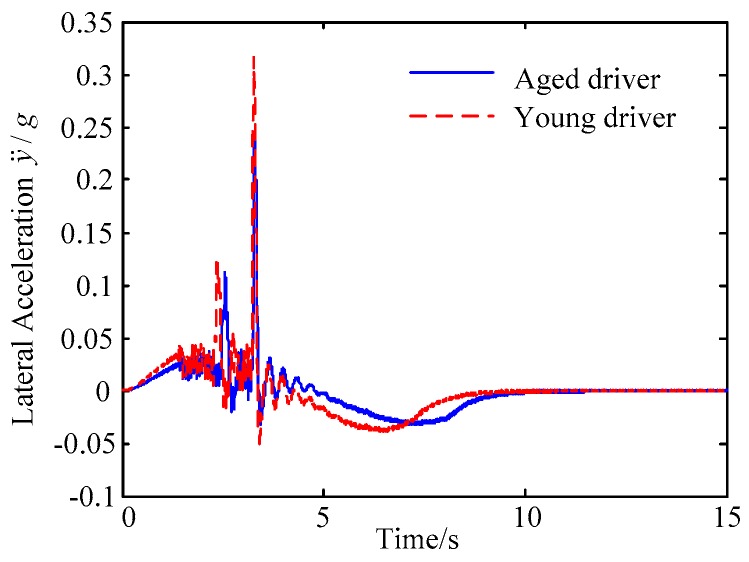
Lateral acceleration of vehicle p under different handling characteristics.

B. Scenario on smaller initial distance. The initial distance is changed to 6.7 m, and the other initial conditions are the same as those of scenario A. The vehicle p is an active vehicle with trajectory re-planning controller. Vehicle q is used as an obstacle vehicle. Figure 19 and Figure 20 are drawn for depicting the real-time trajectories of the two vehicles in lane exchanging. The longitudinal velocities, front wheel steering angle, yaw angle, yaw rate, and lateral acceleration under the different handling characteristics of the driver are shown in Figure 21, Figure 22, Figure 23, Figure 24 and Figure 25.

The discrete points of curves in Figure 19 indicate the position of vehicles at different times. During the entire period of time, vehicle p under different handling characteristics have no collision, and the trajectory is smooth. In this section, vehicle p with young handling characteristics is analyzed for example. In order to give a clearer description of the position of the vehicle when the two vehicles meet, the local position of vehicles is shown in Figure 20. The large rectangles are used to express the contour of the vehicle. The discrete points at the same time are connected by the dotted line and the arrow. There is no collision between vehicles in the process of lane exchanging. As shown in Figure 21, the logic threshold controller selects the speed of vehicle due to the existence of a collision threat under the smaller initial distance. The designed fuzzy PI controller can effectively track the velocity. As shown in Figure 22, Figure 23, Figure 24 and Figure 25, due to the threat of collision, the trajectory re-planning controller needs to re-plan the trajectory to eliminate the collision threat. The peak appears at the moment where the lateral position of the center of mass of the two vehicle approaches. After the peak, with the increase of the lateral distance between the two vehicles, the collision threat is gradually reduced. Due to the coefficient of expansion of the vehicle, the collision threat will take a period of time to completely eliminate. When the collision threat is eliminated, the global controller will track the vehicle trajectory more smoothly, stably and accurately. Due to the different driver characteristics, the vehicle control response is different. Young drivers still maintain the aggressive and sensitive operating characteristics of the steering wheel, making the peak value of the vehicle parameters higher. The lateral acceleration of the vehicle is always less than 0.4g, which ensures the stability of the vehicle.

**Figure 26:**
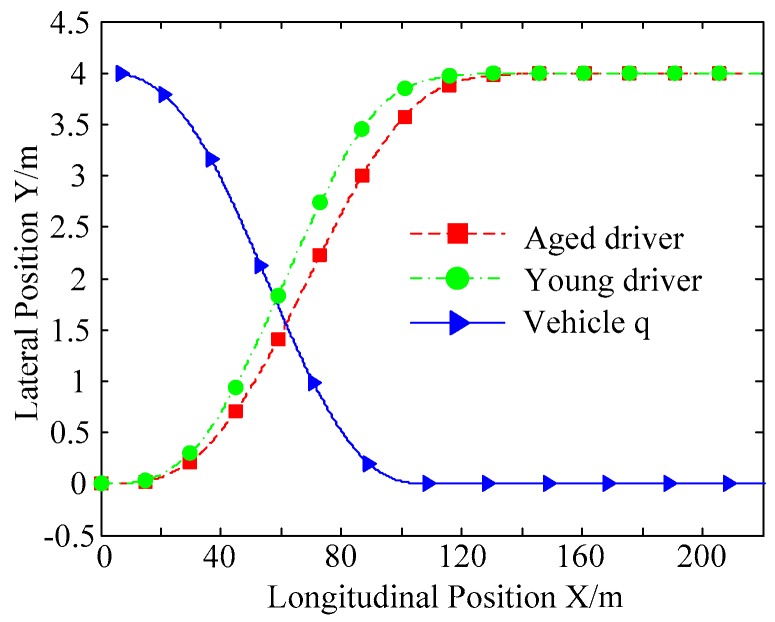
Trajectories of the vehicles driven by different drivers.

**Figure 27:**
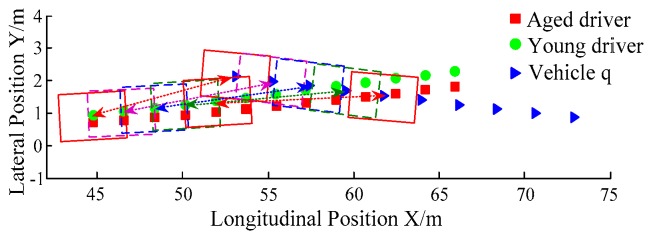
Trajectories of the vehicles driven by different drivers.

**Figure 28:**
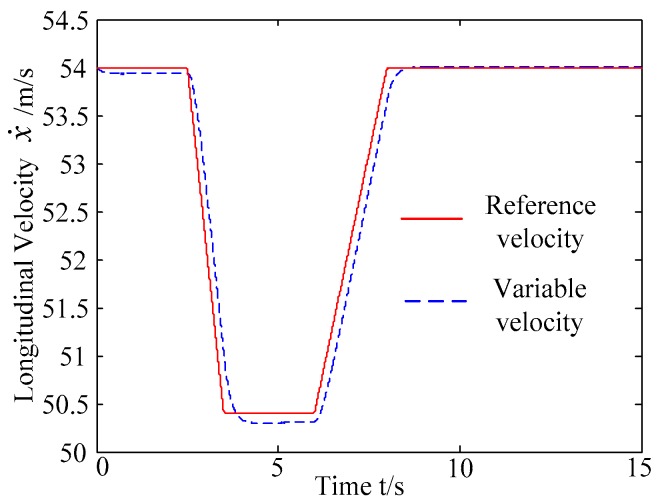
Longitudinal velocities of vehicle p under different velocities of vehicle q.

**Figure 29:**
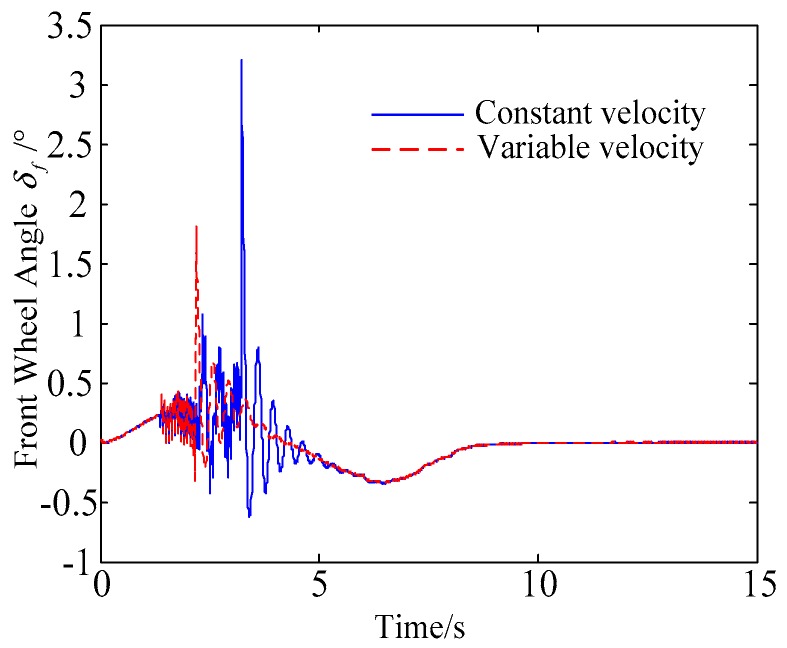
Front wheel angle of vehicle p under different velocities of vehicle q.

**Figure 30:**
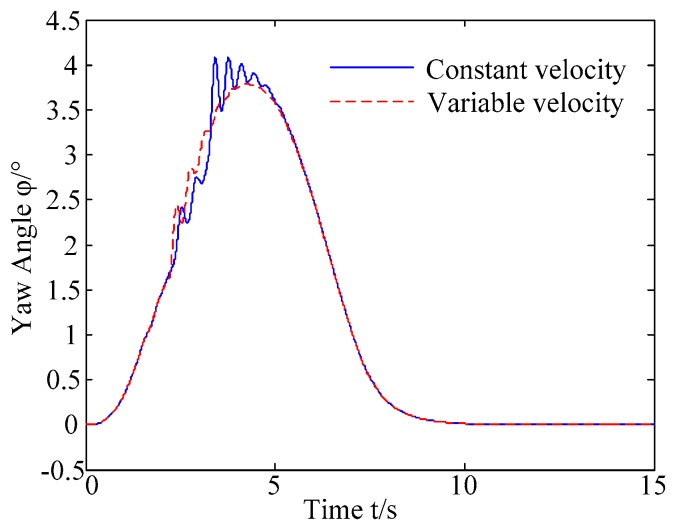
Yaw angle of vehicle p under different velocities of vehicle q.

**Figure 31:**
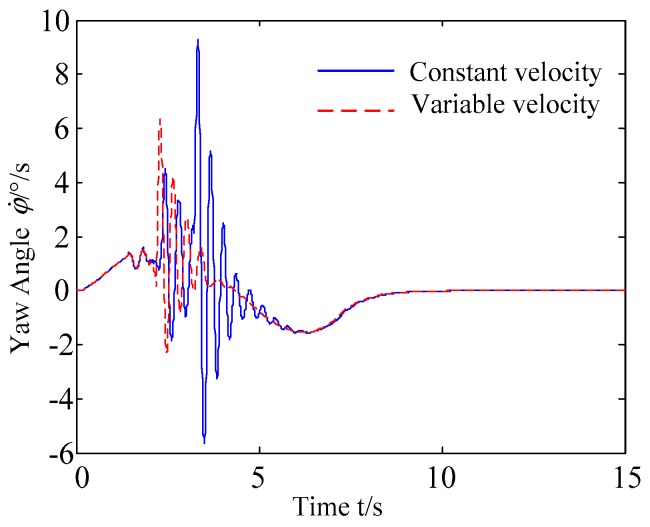
Yaw rate of vehicle p under different velocities of vehicle q.

**Figure 32:**
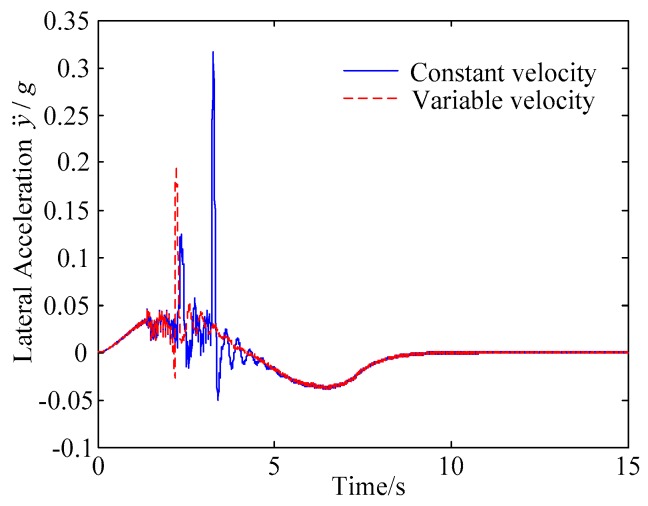
Lateral acceleration of vehicle p under different velocities of vehicle q.

C. Scenario on smaller initial vehicle distance, and variable velocities of vehicle q. In general, vehicle q will increase the velocity to increase the distance between the rear vehicles as much as possible to reduce the collision threat. Based on the scenario B, in this example, vehicle q is set to use the acceleration of 1 m/s^2^ to change lanes, and the controller is simulated and verified. Vehicle p with young handling characteristics is analyzed for example which is more radical and the collision threat is more prominent. Figure 26 and Figure 27 are drawn for depicting the real-time trajectories of the two vehicles in lane exchanging. The longitudinal velocities, front wheel steering angle, yaw angle, yaw rate, and lateral acceleration under the different handling characteristics of the driver are shown in Figure 28, Figure 29, Figure 30, Figure 31 and Figure 32.

The discrete points of curves in Figure 26 and Figure 27 indicate the position of vehicles at different times. During the entire period of time, vehicle p under different handling characteristics has no collision, and the trajectory is smooth. In this section, vehicle p with young handling characteristics is analyzed for example. The large rectangles are used to express the contour of the vehicle. The discrete points at the same time are connected by the dotted line and the arrow. There is no collision between vehicles in the process of lane exchanging. As shown in Figure 28, the logic threshold controller selects the speed of vehicle due to the existence of a collision threat under the small initial distance. The designed fuzzy PI controller can effectively track the velocity. As shown in Figure 29, Figure 30, Figure 31 and Figure 32, because of the collision threat, the trajectory re-planning controller needs to re-plan the trajectory to eliminate the collision threat. In order to track the reference trajectory produced in real time, the MPC controller has frequent fluctuations in the front wheel steering angle, resulting in frequent fluctuations in vehicle yaw angle and yaw rate. Compared with the constant velocity of vehicle q, the acceleration of vehicle makes the longitudinal distance between vehicles increase, and the collision threat between the two vehicles is relatively reduced. Meanwhile, the collision threat will be eliminated faster. The peak value of the front wheel steering angle of vehicle p is relatively reduced. It can reach a steady state faster. The peak value of the yaw angle, the yaw rate, and the lateral acceleration of vehicle p decreases relatively and reach a steady state faster. The lateral acceleration of the vehicle is always less than 0.4 g, which ensures the stability of the vehicle.

The moving obstacle trajectory prediction method proposed in this paper has little effect on the desired trajectory generated by the controller between the vehicles with real-time information interaction. The prediction information produced by the method proposed is more suitable for trajectory information as obstacles. The logic threshold control method can make the appropriate choice for the vehicle velocity and the controller. The local trajectory re-planning controller can adjust the vehicle state parameters and trajectory desired values when the collision threat occurs between vehicles, so that the vehicle can avoid obstacle under the condition of smooth operation.

## 5. Conclusions

In this paper, an integrated avoid collision control framework is proposed for autonomous vehicle trajectory tracking and dynamic collision avoidance. For longitudinal control, a fuzzy adaptive PI controller is proposed for longitudinal velocity tracking. The selection and control of controller and velocity are realized by logical threshold method. For lateral control, a local trajectory re-planning controller based on MPC controller is proposed by collision avoidance control penalty function, which includes many functions such as driver’s handling characteristics, information interaction, real-time obstacle trajectory prediction, local trajectory re-planning.

Simulation results show that the designed controllers can re-plan the real-time trajectory when there is a collision threatening, so as to eliminate the collision conflict. During trajectory re-planning, although the fluctuation of the front wheel steering angle and the states increased, the vehicle parameters are always within the constraints, and the vehicle always maintains stability. The trajectory re-planning motion controller can effectively avoid collision and has good stability.

The time-delay is a key issue in control system [39], especially in autonomous vehicle real-time control. In the future research, the delay problems will be considered in the control framework in order to compensate the delays drawbacks.

## Figures and Tables

**Table 1:** Fuzzy rules of $k_{P}$.

	*ec*	N	Z	P
*e*				
N		N	N	N
Z		N	P	P
P		P	P	P

**Table 2:** Fuzzy rules of $k_{I}$.

	*ec*	N	Z	P
*e*				
N		Z	Z	Z
Z		P	P	P
P		Z	Z	Z

**Table 3:** The main parameters of MPC.

Symbol	Description	Value (Units)
$N_{p}$	Number of prediction points in model predictive control	20
$N_{c}$	Number of control points in model predictive control	5
$s_{1}$	Weight of the lateral offset error of vehicle *p*	$2\times 10^{3}$
$s_{2}$	Weight of the heading error of vehicle *p*	$2\times 10^{4}$
$s_{3}$	Weight of the lateral offset error of vehicle *q*	$2\times 10^{3}$
$s_{4}$	Weight of the heading error of vehicle *q*	$2\times 10^{4}$
r	Weight of the steering control increment	$10^{6}$
$T_{s}$	Sample time of the model predictive controller	0.01 (s)
ρ	Weight of the relaxation factor	1000
ε	Relaxation factor	10
$S_{obs}$	Weight in penalty function	800

**Table 4:** The main parameters of the vehicle.

Symbol	Description	Value (Units)
m	Mass of the vehicle	1530 (kg)
$I_{z}$	Yaw moment of inertia	4607(kg·m^2^)
$l_{f}$	C.g. distance to the front axle	1.11 (m)
$l_{r}$	C.g. distance to the rear axle	1.666 (m)
$C_{cf}$	Front wheel cornering stiffness	1220 (N/deg)
$C_{cr}$	Rear wheel cornering stiffness	1220 (N/deg)
$R_{saf}$	Radius of the safety circle	1.4 (m)
